# Beyond the Appendix Stump: A Rare Case of Appendicular Band Syndrome Causing Small Bowel Obstruction

**DOI:** 10.1155/cris/3114512

**Published:** 2025-10-23

**Authors:** Kumail Jaffry, Amos Nepacina Liew, Niyaz Naqash

**Affiliations:** General Surgery and Acute Surgery Department, Monash Health, Victoria, Australia

**Keywords:** appendectomy, appendicular band syndrome, intestinal obstruction, stump appendicitis

## Abstract

Postoperative adhesions present a complex surgical challenge, often leading to complications, such as small bowel obstruction (SBO). Among these, appendicular band syndrome, although rare, is a serious condition that underscores the importance of meticulous management of the appendix stump during surgery to prevent life-threatening outcomes. We report a case of a 67-year-old female who presented to the emergency department with post-prandial epigastric pain and vomiting. Notably, she did not open her bowels for the last 2 days. The patient had a medical history of hypertension and gastroesophageal reflux disease, and previous surgeries, including laparoscopic appendectomy and ovarian cystectomy. Computed tomography revealed a high-grade, incomplete SBO. Diagnostic laparoscopy revealed thick band adhesions arising from a residual appendiceal stump from previous appendectomy site, which had caused a clockwise torsion of jejunal loops; division of the band and completion appendicectomy resolved the obstruction. These findings highlights the complex interplay between surgical technique and stump length in preventing adhesion formation. The formation of adhesions is primarily initiate from disturbances to peritoneal mesothelial surfaces, triggering inflammatory and coagulation pathways. Our discussion delves into the optimal management of the appendix stump, highlighting current literature that suggests a stump length of approximately 5 mm as optimal for minimising the risk of both stump appendicitis and appendicular band adhesions. While traditional inversion of the stump may limit exposed mucosa, it is not universally recommended because an inverted stump can later mimic a caecal mass or create diagnostic uncertainty. When a laparoscopic endoloop technique is selected, achieving a critical view of the appendix with complete visualisation of the caeco-appendiceal junction before ligation, allows precise placement of the loop flush with the base, thereby keeping the residual stump short and reducing the risk of stump appendicitis. Identifying high-risk patients, with prior abdominal surgery or severe intra-operative inflammation, and tailoring stump management accordingly remain crucial to preventing complications, such as appendicular band syndrome.

## 1. Introduction

Intestinal obstruction accounts for 2%–8% of acute-abdomen presentations, and in roughly 80% of cases the obstruction is located in the small bowel, most frequently due to post-operative adhesions, herniae or neoplasms [1, 2]. Small-bowel obstruction attributable to appendicular band syndrome is exceedingly rare. Appendicular band adhesions are fibrous bands that most often develop after appendicectomy and may encircle or tether adjacent loops of bowel, leading to acute mechanical obstruction [1, 2]. The potential life-threatening nature of this condition underscores the importance of careful appendix-stump management during appendicectomy and justifies detailed reporting of individual cases to guide surgical practice.

## 2. Case Presentation

A 67-year-old female presented with sudden post-prandial epigastric pain, vomiting and 2-day constipation. Past surgery included laparoscopic ovarian cystectomy and appendicectomy 10 years earlier. She was independent with activities of daily living and denied any smoking or alcohol intake.

**Figure 1:**
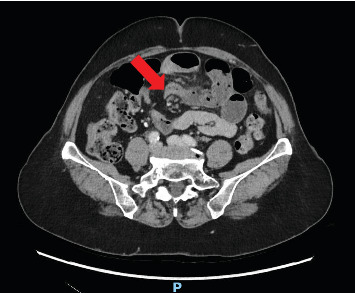
Initial computed tomography of the abdomen and pelvis with contrast showing distended bowel loops with fecalisation, indicative of a high-grade incomplete small bowel obstruction. A single transition point is noted in the lower abdomen, suggesting adhesional cause without evidence of closed-loop obstruction.

On examination, she was diaphoretic with a heart rate of 57 bpm, blood pressure 108/59 mmHg and afebrile. Her abdomen was soft but distended and generally tenderness with sluggish bowel sounds. Routine blood tests including haemoglobin (Hb), white cell count (WCC), C-reactive protein (CRP) and liver function tests (LFT) were normal; lactate 0.9 mmol/L. Contrast CT showed high-grade, incomplete small bowel obstruction (SBO) with a single-transition point in the lower abdomen, without features of closed-loop obstruction or internal hernia, likely adhesional (Figure 1).

**Figure 2:**
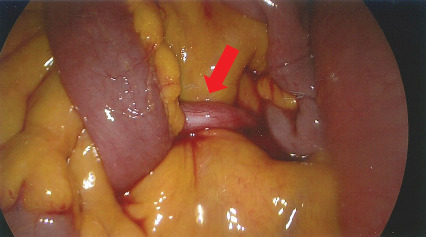
Diagnostic laparoscopy revealing thick band adhesions originating from the stump of a previously removed appendix.

The patient was initially managed with nasogastric decompression and bowel rest. Subsequently, Gastrografin follow-through study showed persistent SBO. Diagnostic laparoscopy showed dense bands arising from a residual appendiceal stump from previous appendectomy site causing clockwise twisting of jejunal loops (Figure 2).

**Figure 3:**
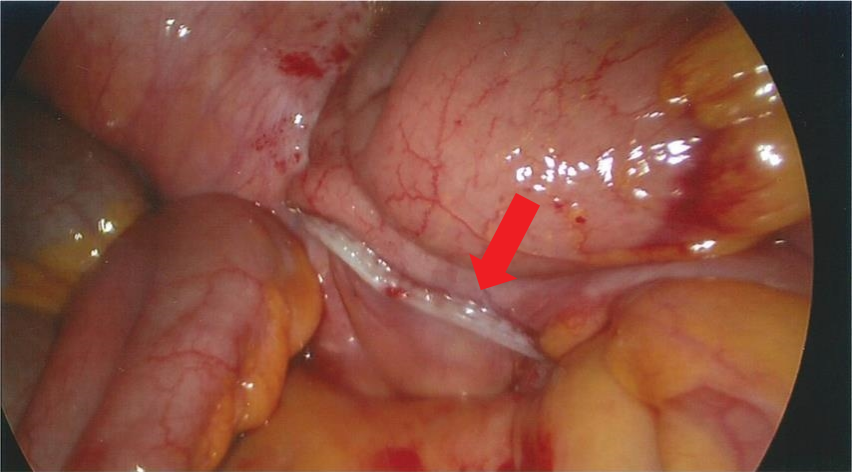
Laparoscopic image post-stapled appendectomy.

Adhesiolysis and stapled completion appendicectomy with EndoGIA60 stapler across the caecal base, preserving the ileocaecal valve, relieved the obstruction (Figure 3). The affected loops of small bowel appeared congested but viable.

Histology revealed a 32 mm × 7 mm appendix remnant with mild transmural eosinophilic infiltrate and an unremarkable 30 mm × 10 mm × 8 mm caecal cuff, consistent with resolving acute appendicitis, without dysplasia or malignancy.

Post-operatively patient received short-term TPN, advanced to a normal diet, mobilised without pain and was discharged fully recovered.

## 3. Discussion

The pathogenesis of adhesion formation following appendectomy is multifactorial, influenced by tissue handling, inflammatory response, surgical technique and individual patient factors, such as prior infections or surgeries [1–3]. The formation of adhesion is thought to be triggered by disturbances to the peritoneal mesothelial surfaces, activating coagulation and inflammatory pathways [1, 3]. These surfaces are particularly prone to forming adhesive bands when injured, as part of the body's healing response. Macrophages attract mesothelial cells to heal the area, but adhesions often occur due to an imbalance in fibrin breakdown and absorption, leading to fibrous bands that can cause complications, such as SBOs [1, 3].

In our case, the patient presented with a SBO secondary to dense fibrous bands originating from the long residual stump of a previous appendectomy—an example of appendicular band syndrome [1, 2]. Early operative exploration is key: diagnostic laparoscopy and converting to open if exposure is inadequate permits safe adhesiolysis, completion appendicectomy and rapid assessment of bowel viability, with resection reserved for clearly ischaemic segments [2, 4]. Post-operatively, careful resuscitation, gradual dietary upgrade and vigilance for ileus are essential to optimise recovery.

Following appendectomy, the optimal length of this stump has been debated, with concerns focusing on the risk of stump appendicitis and adhesions leading to bowel obstruction [5, 6]. Stump appendicitis, a rare but recognised complication following appendectomy, occurs when a remnant of the appendix becomes inflamed. Similarly, excessively long stumps have been associated with an increased risk of adhesions, which may lead to bowel obstructions as seen in our case [6, 7].

Although there is ongoing debate about whether traditional ‘complete' appendectomy (with stump imbrication) reduces adhesion-related complications than endoloop approaches, large comparative studies remain limited. Likewise, data on stapled appendectomies causing adhesion-related complications are scarce, and any potential benefits must be weighed against the increased cost of specialised equipment.

Traditional complete appendectomies, in which the appendiceal stump is often inverted and buried (imbricated) within the caecum, are theorised to reduce exposed mucosa and potential nidus for stump inflammation or band formation [8, 9]. Some reports suggest that burying the stump may help limit direct contact between the residual appendix and adjacent bowel loops, potentially lowering adhesion formation [9, 10]. In contrast, endoloop approaches, though safe, minimally invasive and quick, can often leave a longer stump, especially when the mesoappendix is thick or when the surgeon opts to place the loop loose and at a distance from the base of the appendix to avoid cutting through swollen base of the appendix [9, 10]. This longer remnant could, in theory, elevate the risk of stump appendicitis or serve as a focal point for fibrous band formation.

Several technical refinements help mitigate this risk when an endoloop approach is employed. Critical view of the appendix: the mesoappendix is fully skeletonised, the caeco-appendiceal junction is clearly visualised and gentle traction–counter-traction is applied allowing for precise placement of the endoloop at the true base. These manoeuvres can minimise residual stump length and reduce the likelihood of stump appendicitis [1, 11].

Nevertheless, inversion is not without drawbacks and is, therefore, not routinely practised. An inverted, ligated stump can resemble a caecal tumour on imaging or during surgical exploration, prompting unwarranted investigation or resection [12, 13]. Furthermore, excessive inversion may distort the caecal wall and compromise blood supply; when inversion is deemed necessary, most authors now advocate a Z-plasty closure rather than a purse-string suture, particularly for unligated stumps to reduce the risk of luminal narrowing or serosal tearing [12, 13].

To date, high-level evidence comparing these two techniques (complete imbrication versus endoloop) remains limited. Most available data are from small case series or retrospective studies, which offer observational rather than high-level evidence. For instance, some authors report fewer cases of stump appendicitis with shorter or inverted stumps, whereas others highlight that extremely short stumps can lead to tension or devascularisation issues [14, 15]. Thus, maintaining an optimal stump length appears crucial for minimising both stump appendicitis and adhesional complications.

In addition to surgical technique, certain patients may be at higher risk for post-operative adhesion formation. These include active peritonitis at the time of surgery, delayed presentation of acute appendicitis especially with perforation or abscess and a history of multiple abdominal surgeries which can predispose to more robust inflammatory responses and subsequent fibrous band development [16]. Comorbidities, such as poorly controlled diabetes, obesity or immunosuppression can further compromise wound healing and promote adhesion formation. Perhaps, recognising these high-risk profiles could influence intraoperative decisions—surgeons might opt for more careful stump management with imbrication, minimal handling of tissues in these patients.

The current trend toward minimally invasive appendectomy (laparoscopic, endoloop, Hem-o-lok or stapled) provides clear benefits in terms of reduced postoperative pain and recovery time. However, as illustrated by this case, surgeons must remain vigilant about potential late complications linked to longer or inadequately managed stumps. While no consensus firmly dictates that complete appendectomy with stump burial universally prevents appendicular band formation, adopting meticulous surgical technique and aiming for an optimally sized stump—often cited as approximately 5 mm—may help reduce the risk of stump appendicitis and adhesion-related complications [6, 16–18]. Future large-scale prospective studies could help clarify whether traditional imbrication strategies indeed yield relatively lower rates of appendicular band syndrome.

In conclusion, our case underscores that appendicular band syndrome remains a rare but significant cause of SBO, especially when the appendiceal stump is left long enough to form adhesive bands. Surgeons should individualise their approach to stump management based on patient risk factors, intraoperative findings and familiarity with specific techniques, balancing the goal of preventing stump appendicitis while also minimising the likelihood of adhesion complications.

## Data Availability

The data sharing is not applicable to this article as no new data were created or analysed in this study.
